# A Rare Case Report on Suboccipital Region Benign Giant Osteoma

**DOI:** 10.1155/2016/2096701

**Published:** 2016-03-09

**Authors:** Sunil Munakomi, Binod Bhattarai

**Affiliations:** Department of Neurosurgery, College of Medical Sciences, P.O. Box 23, Chitwan, Nepal

## Abstract

Herein we report a rare case of a giant suboccipital osteoma in a 55-year-old woman presenting primarily due to cosmetic issue. We discuss the management algorithm taken in the patient, highlighting excision of a potentially curable bony tumor only after ruling out its extension to the ear cavity, mastoid ear cells, transverse sinus, and the intracranial compartment.

## 1. Introduction

Osteomas in the occipital and mastoid regions are exceptionally rare with only 137 cases reported in the literature [1–3]. Asymptomatic in most of the cases, patients may present with esthetic issues or symptoms of external auditory obstruction [1, 4]. Computed tomography is the gold standard for diagnosis [5]. The main aim of the radio imaging is to rule out invasion of the inner table of the calvarium and its intracranial extension of the lesion [1]. Complete excision in the symptomatic and giant osteomas is the therapeutic goal [4].

## 2. Case Report

**Figure 1:**
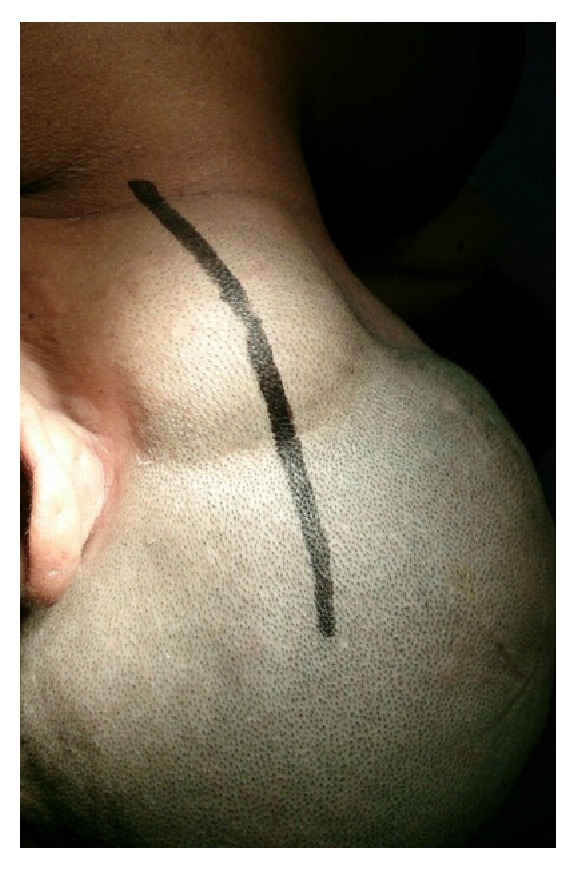
Image showing the extent of lesion and the planned surgical incision mark.

**Figure 2:**
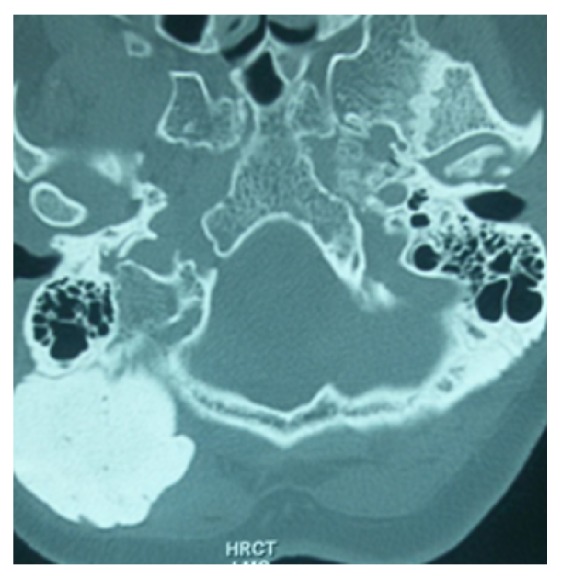
CT bone window showing the large lesion in the right suboccipital and adjacent mastoid region.

**Figure 3:**
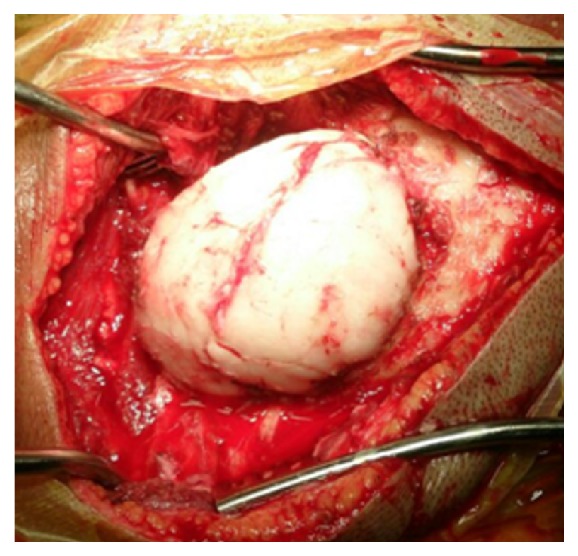
Intraoperative picture outlining the lesion.

**Figure 4:**
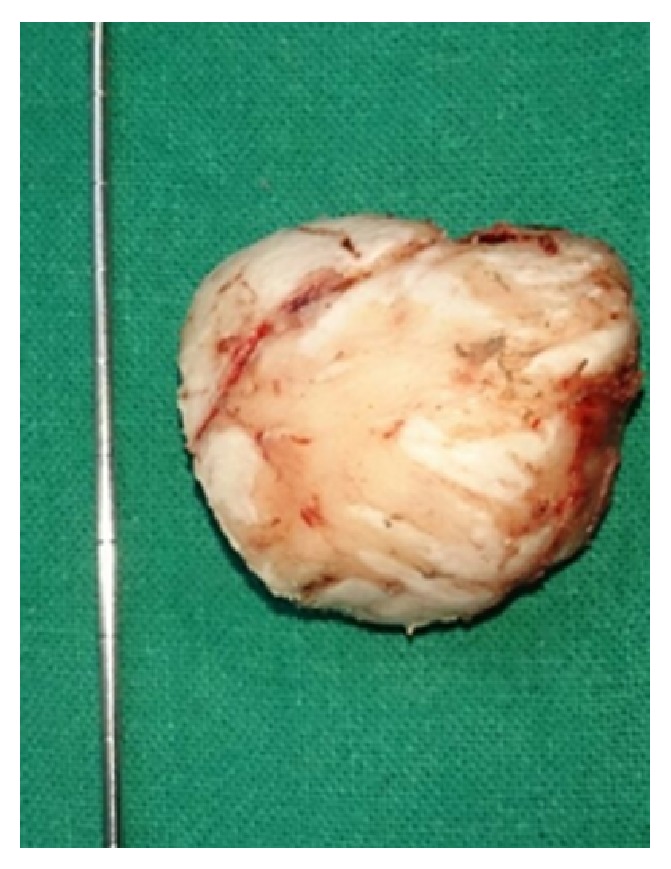
The excised bony lesion.

**Figure 5:**
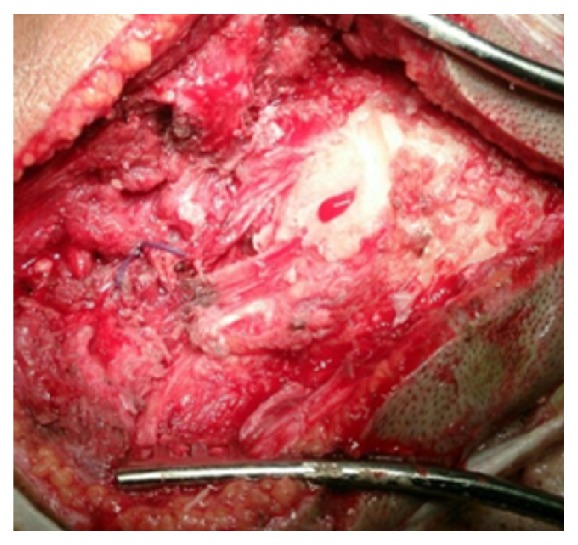
The calvarial base after excision of the bony tumor.

A fifty-five-year-old woman from Chitwan, Nepal, visited our patient surgical outpatient clinic with a chief complaint of slowly progressive swelling at the back of her head. She had detected the swelling since her twenties. There was no history of trauma, redness, ear discharge, deafness, or similar swellings elsewhere in her body. It slowly progressed in size over time. Once it attained a massive size, she sought medical advice. There were no important past medical or surgical illnesses. Her bladder and bowel habits were normal. Examination revealed a bony and sessile swelling on the right suboccipital region and extending below the craniovertebral junction (Figure 1). The skin overlying the lesion was normal. The margin of the lesion was clearly demarcated. The cranial nerves were intact. The otoscopic examination was normal. CT scan revealed a hyperostotic spherical lesion measuring 6 × 5 cm^2^ within the right suboccipital region highly suggestive of a giant osteoma (Figure 2). Because of the large size and primarily for cosmetic reasons, she was counseled for surgical excision of the lesion. The lesion proved to be a bony sessile mass extending from the lambdoid suture superiorly to C1 arch inferiorly (Figure 3). The lesion was excised with the assist of a Gigli saw and later chiseled (Figure 4). The bleeding from the base was controlled with the application of a bone wax. The mastoid air cells were not violated. The inner table of the bone beneath was intact (Figure 5). The postoperative period of the patient was uneventful and she was discharged on the third day. The histopathological study confirmed the compact variant of benign osteoma. The patient followed up in the OPD 2 months later. The scar was healthy and she had no new complaints. She was assured and advised for a six-month follow-up.

## 3. Discussion

Osteoma is a slow-growing benign mesenchymal osteoblastic tumor formed by mature bone tissue [6]. Osteomas, constituting 0.1–1% of all benign skull tumors, are extremely rare [7]. The most common site reported is the frontoethmoidal region and neighboring sinuses. Involvement of the temporal and occipital squama is extremely rare [8]. Osteomas larger than 3 cm are termed giant osteomas [9]. They are also common in the frontoethmoidal region with above 40 cases reported in the literature [10, 11]. Only few cases of giant osteomas involving the occipital region [2, 3], posterior skull base [12], and the atlas [4] have been reported in the literature so far.

Etiology of the entity includes trauma, previous surgery, radiotherapy, chronic infection, and hormonal factors [13]. They may be a reliable marker for early detection of carriers of Gardner syndrome [14]. They are mostly asymptomatic, but they can present with deformity, swelling, pain, deafness, and chronic discharge [15]. Computed tomography is the imaging modality of choice which demonstrates a rounded bony lesion on the mastoid outer cortex having distinctive margins with sessile or pedunculate base [16, 17].

The main differential diagnosis includes osteosarcoma, osteoblastic metastasis, isolated eosinophilic granuloma, ossifying fibroma, Paget's disease, giant cell tumor, osteoid osteoma, hemangioma, calcified meningioma, and monostotic fibrous dysplasia [5, 18–21]. However, edges of these lesions are generally less distinct compared to the osteomas.

Osteomas are resected only if they are symptomatic or else for cosmetic reasons. The surgical target must be outlining normal cortical bone all around the lesion. Because these lesions are limited to the external cortex, finding a plane of cleavage between the osteoma and normal bone is not difficult [22]. If mastoid air cells are exposed, a cortical mastoidectomy should be done [23]. Partial excision is justified if there is an extension to either facial nerve, bony labyrinth, or the fallopian canal [24, 25]. In such invasive scenario, damage to the facial nerve, tearing of the sigmoid sinus, and postoperative auricular discharge may complicate the postoperative course [13].

Histologically, osteomas are composed of well-differentiated, mature bone characterized by dense lamellae with organized Haversian canals. Histologically, there are three different subtypes: compact, spongiotic, and mixed [25].

The prognosis of the osteoma may be considered the best in terms of cosmetic and curative aspects provided complete excision is undertaken. Malignant transformation has not been reported yet [24]. The recurrence is also uncommon as only two cases have been reported so far [26].

In young patients with skull osteomas, complete workup needs to be done to rule out Gardner syndrome by screening for the concurrent presence of intestinal polyps, soft tissue tumors, and dental abnormalities [27].

## 4. Conclusion

Giant occipital osteomas have been rarely reported in the literature. Like giant osteomas in other locations of the skull, they can reach large volumes but are essentially benign and potentially curable by excision. Proper assessment of its extension especially when it is in the vicinity of the mastoid and the suboccipital regions is imperative to providing complete excision and limiting postoperative complications.
